# Interstitial Lung Disease in Rheumatoid Arthritis: A Review

**DOI:** 10.7759/cureus.53632

**Published:** 2024-02-05

**Authors:** Shahad Al-Baldawi, Gabriel Zúñiga Salazar, Diego Zúñiga, Sneha Balasubramanian, Khawar Tariq Mehmood

**Affiliations:** 1 Department of Rheumatology, Al-Yarmouk Teaching Hospital, Baghdad, IRQ; 2 Department of Medicine, Universidad Católica de Santiago de Guayaquil, Guayaquil, ECU; 3 Department of Internal Medicine, Madras Medical College, Chennai, IND; 4 Department of Internal Medicine, Aster Hospital Br of Aster Dm Healthcare FZC, Dubai, ARE

**Keywords:** rheumatoid arthritis, interstitial lung disease in rheumatoid arthritis, rheumatoid arthritis lung complications, interstitial lung disease treatment, pulmonary involvement in rheumatoid arthritis, interstitial lung disease

## Abstract

Rheumatoid arthritis (RA) is a chronic inflammatory autoimmune disorder. Although the joints are typically the first area affected in RA, it can also involve extra-articular regions. This article provides an overview on rheumatoid arthritis-associated interstitial lung disease (RA-ILD), a component of the disease manifestations leading to significant morbidity and mortality. Managing these pulmonary symptoms in people with RA poses a number of difficulties for medical professionals. In this review article, we shed light on the prevalence of RA-ILD and the common pulmonary manifestations of RA, while focusing on the evolving pathogenesis concepts that link them to RA's autoimmune cascade. We also address the diagnostic challenges and the available screening modalities that aid in the early recognition and effective management of these pulmonary complications. Furthermore, glucocorticoids, disease-modifying antirheumatic medications, immunosuppressive medications, and biological agents are among the pharmacological approaches that have been explored in this review study.

## Introduction and background

Rheumatoid arthritis (RA) is a chronic systemic inflammatory disease that can result in irreversible changes to the joints, causing critical disability and poor quality of life. Diagnosis is made by a combination of both clinical and laboratory features. Around 1.3 million people in the United States have RA, representing 0.6% to 1% of the population [1]. The precise cause of RA is unknown; in any case, it appears to result from the interaction between genetic liabilities and environmental factors [2]. Females, smokers, and those with a family history of the illness are most frequently affected [3]. Extra-articular manifestations of the disease can occur in roughly 50% of the affected patients, with the most common site of involvement being the lung [4]. Whereas any part of the lung can be affected, rheumatoid arthritis-associated interstitial lung disease (RA-ILD) is the driving cause of death in patients with RA, leading to significant morbidity and mortality [5]. While it can be the initial presenting manifestation in 10% to 20% of patients, most RA-ILD occurs within the first five years after diagnosis [6]. RA-ILD can present clinically with a wide range of symptoms, the most common of which are dyspnea and cough [7]. Histopathological analysis is the most valuable in classifying and differentiating the different types of interstitial lung disease (ILD), which can be challenging to differentiate [8]. Additionally, high-resolution CT (HRCT) is the gold standard tool for classifying ILD [9]. Current studies highlight that RA-ILD patients may have up to a three-fold chance of mortality compared to those without ILD. Even though the overall mortality from RA is diminishing, mortality from RA-ILD is rising, especially in women and in the elderly population [10,11]. They are raising concerns regarding early diagnosis and effective management of RA-ILD. In this review article, we shed light on the classifications, pathophysiology, and manifestations of RA-ILD, vocalize the possibilities and advantages of screening and early diagnosis, as well as discuss clinical and therapeutic approaches, including immunomodulating and anti-inflammatory drugs, such as biological and disease-modifying antirheumatic drugs (DMARDs).

## Review

RA may affect any portion of the lung; it can involve the parenchyma showing as ILD, or it can affect the pleura, leading to pleural inflammation and effusions, small or large airways manifesting as cricoarytenoiditis, constrictive or follicular bronchiolitis, and bronchiectasis. Lastly, it can involve the lung vessels causing vasculitis or pulmonary hypertension [5].

Interstitial lung disease

**Figure 1:**
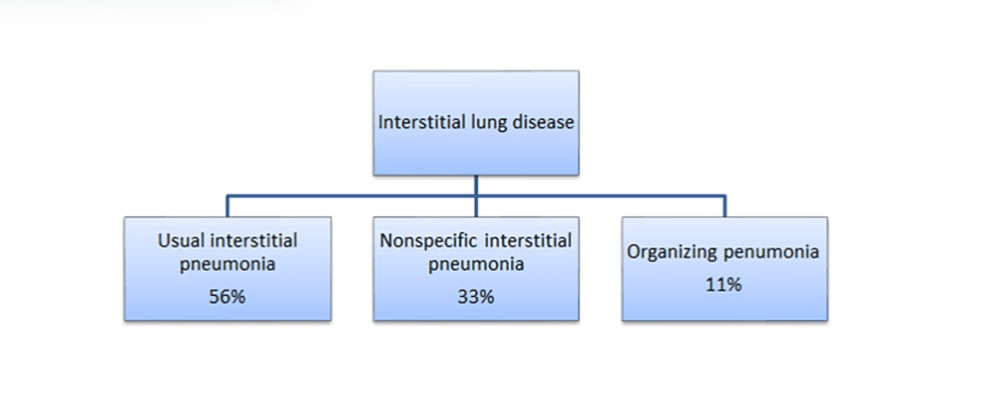
Common classification of interstitial lung disease in rheumatoid arthritis. Data retrieved from [9].

Estimates of prevalence and other general features of RA-ILD vary widely. Several histopathological patterns of RA-ILD have been described. Usual interstitial pneumonia (UIP) is the most frequent one, followed by nonspecific interstitial pneumonia (NSIP); other patterns are seen less frequently [12]. The frequency of RA-ILD forms is summarized in Figure 1. The consensus classification for idiopathic interstitial pneumonias (IIPs) has been used to define RA-ILD, as there is no specific categorization for the condition [13]. Acute interstitial pneumonia, diffuse alveolar damage, organizing pneumonia (OP), desquamative interstitial pneumonia, lymphocytic interstitial pneumonia, and others have also been identified in patients with RA [14,15]. On HRCT, UIP is characterized by honeycombing that appears as clustered cystic airspaces, which are usually 3-10 mm in diameter but can infrequently reach 2.5 cm. It has clearly defined walls and is typically subpleural [16]. According to biopsy findings, NSIP is characterized by diffuse alveolar septal thickening with lymphoplasmacytic inflammation and accentuation around bronchioles.

Pathophysiology and risk factors

The pathophysiology of RA-ILD still needs to be better understood. Genetics and environmental factors are critical in how ILD develops in RA patients [17]. Some significant human leukocyte antigen (HLA) variations that can contribute to the emergence of ILDs in RA patients include HLA-DRB1, HLA-DR4, and HLA-B40 [18]. Smoking has been shown to interact with HLA-DR shared epitope (SE) genes and play a significant role in initiating the immune response to citrulline-modified proteins [19]. Lung parenchymal and airway damage due to exposure to environmental factors can increase protein citrullination in lung cells [20]. In a genetically susceptible person, the pathological process starts with an inflammatory process that activates cytokines, chemokines, and growth factors such as tumor necrosis factor (TNF), interleukins (IL), and vascular endothelial growth factor (VEGF) [21]. Matrix metalloproteinases (MMP) become hyperactive, and extracellular matrix (ECM) is deposited more readily as a result of fibroblast proliferation and differentiation, which leads to the onset of pulmonary fibrosis and ILD [5]. Recent research revealed that the role of IL-17 in the development of pulmonary fibrosis in mice with RA-ILD and idiopathic pulmonary fibrosis (IPF) may have clinical implications for treatment strategies targeting pulmonary fibrosis in both conditions. Transforming growth factor-1 and other TH17 cytokines, like IL-17A, stimulate fibroblast proliferation and ECM production, contributing to fibrosis development [22,23]. There are significant variances between IPF and RA-ILD even though both conditions share immunological pathways. Patients with RA-ILD have more inducible bronchial-associated lymphoid tissue in their lung tissue than do those with IPF, indicating that immunological dysregulation may affect RA-ILD more so than IPF [24].

The assessment of risk factors is crucial for RA-ILD due to the disease's relevant prevalence as well as the effect that the diagnosis has on mortality rates and treatment options [25]. A cross-sectional study was done by Juge et al. on patients who met either the American College of Rheumatology (ACR) 1987 classification criteria for RA or the 2010 ACR/European Alliance of Associations for Rheumatology (EULAR) classification criteria for RA. Results have identified independent risk factors for RA‐ILD; compared with patients with RA who do not have ILD, those with RA‐ILD more frequently carried the MUC5B rs35705950 T risk allele, were more frequently men, were older at the onset of RA, and had higher DAS28-ESR (Disease Activity Score in 28 joints using the erythrocyte sedimentation rate) scores. Patients with RA-ILD also exhibited higher mean C-reactive protein levels during follow-up, higher body mass index (BMI), and longer duration of tobacco use (pack years) [26]. Another prospective cohort study conducted in Boston, Massachusetts, including 1419 RA patients who started enrolling subjects in 2003, concluded that the level of disease activity was directly associated with an increased risk of developing RA-ILD, Accordingly, reducing systemic inflammation is hypothesized to alter the course of RA-ILD development [27].

Diagnosis and screening

A multidisciplinary approach, typically comprising pulmonologists, radiologists, and pathologists, is necessary for the differential diagnosis of ILDs [28]. A summary of the 2023 ACR guidelines for the screening of ILD in people with systemic autoimmune rheumatic diseases (SARDs) is included in Table 1. The gold standard method for detecting ILD in RA patients was found to be HRCT, considering its extreme sensitivity [29]. A subpleural, basal predominance, reticular abnormality with honeycombing, traction bronchiectasis, a relative lack of ground-glass opacities, and air trapping on exhale are the hallmarks of the UIP [30]. HRCT can be used to predict the increasing fibrosis of ILD associated with RA in some cases. A retrospective study published in 2019 concluded that a wide distribution of subpleural reticular pattern (RP) and/or interlobular septal thickening and peribronchovascular interstitium (PBVI) thickening on HRCT shows predictivity of progressive fibrosis in RA-ILD [31]. In 2014, a prospective study was done in Switzerland on 205 systemic sclerosis (SSc) patients, suggesting that a specific, reduced HRCT procedure with nine slices distributed according to a basal-apical gradient is a reliable and accurate way to identify ILD in SSc patients. This study proves that it can be incorporated into daily clinical routine for early diagnosis and screening of ILD, with the significant benefit of a low radiation dosage compared with the standard whole-chest HRCT [32]. In clinical practice, multiple pulmonary function tests (PFTs) are often considered when assessing the presence of lung restriction as in RA-ILD; however, it was found that when depending merely on PFTs, there is a considerable chance of missing major SSc-related ILD [33]. There is some research on using transthoracic lung ultrasonography for checking impending pulmonary structural alterations in RA patients. In a study done by Moazedi-Fuerst et al. on 64 consecutive patients with RA and 40 healthy volunteers, 28% of RA patients showed pleural nodules or B-line phenomena. In these patients, CT scans showed signs of incipient ILD [34]. As for monitoring for ILD progression, the 2023 ACR guidelines recommend using HRCT chest and/or PFTs. Using both HRCT chest and PFTs is preferred over using PFTs alone. For people with RA-ILD, it is suggested to use PFTs for monitoring every three to 12 months rather than shorter or longer intervals, for the first year, then less frequently once stable [35]. A summary of studies showing some of the risk factors and diagnosis modalities in RA-ILD is included in Table 2.

**Table 1:** The 2023 American College of Rheumatology (ACR) guidelines for the screening of ILD in people with systemic autoimmune rheumatic diseases. PFTs: pulmonary function tests; SARDs: systemic autoimmune rheumatic diseases; ILD: interstitial lung disease; HRCT: high-resolution computed tomography; 6MWD: 6-minute walk test distance. Data retrieved from [35].

Screening modality	ACR recommendations
Pulmonary function test	Conditionally recommend screening with PFTs for people with SARDs at increased risk of developing ILD.
High-resolution CT scan	Conditionally recommend screening with HRCT of the chest for people with SARDs at increased risk of developing ILD.
6-minute walk test distance	Conditionally recommend against screening with 6MWD chest for people with SARDs at increased risk of developing ILD.
Chest radiography	Conditionally recommend against screening with chest radiography for people with SARDs at increased risk of developing ILD.
Ambulatory desaturation testing	Conditionally recommend against screening with ambulatory desaturation testing for people with SARDs at increased risk of developing ILD.
Bronchoscopy	Conditionally recommend against screening with bronchoscopy for people with SARDs at increased risk of developing ILD.
Surgical lung biopsy	Strongly recommend against screening with surgical lung biopsy for people with SARDs at increased risk of developing ILD.

**Table 2:** Overview of the included studies. RA: rheumatoid arthritis; RA-ILD: rheumatoid arthritis-associated interstitial lung disease; HRCT: high-resolution computed tomography; SSc: systemic sclerosis; SSc-ILD: systemic sclerosis-associated interstitial lung disease; DAS28-ESR: Disease Activity Score in 28 joints using the erythrocyte sedimentation rate.

References	Publication year	Type of the study	Population	Conclusion
Juge et al. [26]	2022	Cross-sectional study	The discovery population incorporated 163 patients with RA, and the replication population incorporated 89 patients with RA.	Findings suggest that patients with RA‐ILD more frequently (1) carried the MUC5B rs35705950 T risk allele; (2) were more frequently men; (3) were older at the onset of RA; (4) had higher DAS28-ESR and higher mean C-reactive protein levels; (4) had a higher body mass index; (5) had a longer duration of tobacco use (pack years).
Sparks et al. [27]	2019	Prospective cohort study	1,419 participants at Brigham and Women’s Hospital in Boston; all subjects have RA according to the treating physician and accepted criteria.	Active RA is a risk factor for the emergence of RA-ILD. In contrast to remission/low disease activity, high/moderate disease activity was linked to a two-fold increased risk of RA-ILD.
Li et al. [31]	2019	Retrospective study	1096 RA patients, 213 of whom had a diagnosis of RA-ILD underwent serial chest HRCT.	Increasing fibrosis in RA-ILD appears to be more likely predicted by HRCT abnormalities than by other criteria.
Frauenfelder et al. [32]	2014	Prospective study	A total of 205 consecutive patients with a diagnosis of SSc and annual follow-up in the Department of Rheumatology, University Hospital Zurich.	Reduced chest HRCT procedure has the advantage of a significantly lower radiation dose compared to traditional whole-chest HRCT, and it reliably detects even moderate SSc-ILD in clinical practice.
Moazedi-Fuerst et al. [34]	2014	Prospective study	64 consecutive patients with rheumatoid arthritis and 40 healthy volunteers.	Transthoracic ultrasound of the lung is an affordable and secure method for screening RA patients for developing pulmonary structural abnormalities.

Treatment

Immunosuppressants

The effect of immunosuppressants on typical interstitial pneumonia (UIP) in RA or connective tissue disease (CTD) is unclear. However, some retrospective research has revealed that immunosuppressants perform better in ILD types other than UIP. Thus, immunosuppressive medication might be more effective on RA-ILD with NSIP or OP patterns than on the UIP pattern [13,36]. However, a lung biopsy can also reveal varied histologic abnormalities in different specimens, and some individuals have unclassifiable or mixed patterns on HRCT. Unfortunately, not much data exists that explains how well the medication was performed and how the condition progressed in these patients over time [37].

Given their effectiveness in CTD-ILD rather than RA-ILD, glucocorticoids are frequently included in the first therapy regimen for clinically significant RA-ILD [38]. NSIP and OP ILD patterns are more likely to respond to glucocorticoids than UIP [38]. In a retrospective case series published in 2018 on 26 ILD patients with underlying CTD diagnoses, prednisone and oral tacrolimus were administered after two courses of pulse-dose methylprednisolone therapy, which seemed to be well tolerated and to have multifaceted efficacy [39]; on the contrary, there is no evidence to support a role for steroids as monotherapy in IPF. Mayo Clinic investigators presented a retrospective intent-to-treat study of survival for 157 IPF patients receiving no medication, 54 patients receiving maintenance doses of prednisolone alone, 167 patients receiving colchicine alone, and 71 patients receiving both colchicine and prednisolone. There was no statistically significant difference in survival between individuals receiving prednisolone medication and those not receiving any treatment after adjusting for age, sex, and lung function [40]. Corticosteroids raised the risk of life-threatening infections in RA-ILD patients. Despite the use of DMARDs, It was discovered that a higher frequency of infections was associated with a mean daily dose of prednisone greater than 10 mg [41]. As such, their best use is in the early management of acute exacerbations or their treatment until new medicines with better long-term safety profiles are introduced.

Other immunomodulatory treatments such as mycophenolate mofetil (MMF), cyclophosphamide, azathioprine, cyclosporine, and tacrolimus may also be used to treat RA-ILD, but their effects are still unclear. MMF was well tolerated in a large, heterogeneous cohort of 125 CTD-ILD patients (18 with RA-ILD), and it had a low discontinuation rate. Over a median follow-up of 2.5 years, MMF therapy was linked to either stable or better pulmonary physiology [42].

Despite the lack of controlled clinical trials for cyclophosphamide in RA-ILD and a lack of efficacy data, it continues to be used in clinical practice [43], especially in cases of ILD that are highly progressive. In 2019, a retrospective study examined the variables linked to progression and survival in 266 RA-ILD patients, finding that those receiving cyclophosphamide treatment had a better prognosis [44]. In RA-ILD, azathioprine is frequently used as an alternative to methotrexate. According to a single-center retrospective cohort analysis of CTD-ILD, azathioprine patients experienced comparable clinical events and longitudinal PFTs compared to MMF patients (n = 97, 24% RA-ILD) [45]. Although these additional immunomodulatory treatments (such as MMF and azathioprine, for example) may successfully treat ILD, healthcare professionals must consider the possibility of increased side effects and less favorable effects on articular disease [46,47].

Conventional Disease-Modifying Antirheumatic Drugs (cDMARDs)

Only 0.3% to 0.4% of RA patients using methotrexate develop pneumonitis [48]. Moreover, methotrexate does not increase the risk of RA-ILD. Results from prospective early RA inception cohorts indicated a tendency for RA patients on methotrexate to have a lower likelihood of developing ILD (odds ratio: 0.54; 95% CI: 0.28-1.06) [49]. A study done by Rojas-Serrano et al. published in 2017 including 78 patients observed a prolonged survival (HR: 0.13, 95% CI: 0.02-0.64) in RA-ILD patients receiving MTX compared to patients receiving other cDMARDs after adjusting for confounding variables [50]. The avoidance of methotrexate in patients with or at risk for RA-ILD may be partly responsible for the higher rate of ILD observed with leflunomide treatment [51]. Leflunomide should be either avoided or used with caution in patients with prior methotrexate pneumonitis and in patients with pre-existing ILD [52]. They indicate that in these situations, it should not be the substitute for methotrexate. Pneumonitis has also been documented with sulfasalazine use [53]. Data on the safety of hydroxychloroquine in RA-ILD are limited.

*Biological Disease-Modifying Antirheumatic Drugs (bDMARDs* *)*

Anti-tumor necrosis factor (anti-TNF) agents have demonstrated excellent effectiveness in slowing the advancement of articular disease and symptoms. However, warnings about possible pulmonary toxicity have come up as a result of its growing use [54]. All anti-TNF drugs approved for RA have been correlated with new-onset or worsening of existing ILD: infliximab [55], etanercept [56], and adalimumab [57]; also, some of the newer agents, including certolizumab [58] and golimumab [59]. Other investigations disproved the lung toxicity of TNF inhibitors (TNFi) and demonstrated that these agents have the potential for stabilizing or possibly improving pulmonary interstitial disease [60-62]. The British national prospective observational trial of 367 individuals with pre-existing RA-ILD found that treatment with TNFi did not increase mortality in patients with RA-ILD when compared to cDMARDs; however, the percentage of RA-ILD-related mortality was higher (34%) among patients receiving TNFi therapy [63]. In a longitudinal observational trial involving 263 patients with RA-ILD, it appears that abatacept (ABA) is similarly beneficial in stabilizing dyspnea, lung function, and radiological deterioration in both UIP and NSIP patterns of the disease. Regardless of the radiological pattern, early ABA administration may stop the progression of RA-ILD [64].

Researchers have found that 10% of patients experienced ILD following the introduction of anti-TNF agents in a case series of 226 patients taking anti-TNF drugs (of whom 83% had RA) [65]. Interestingly, anti-TNF-induced ILD has a significant mortality rate of one-third of patients, which rises to two-thirds in patients who already have ILD [65,66]. Anti-TNF therapy should be used cautiously in patients who already have RA-ILD. Other factors that increase the possibility of death include advanced age (>65 years), a later diagnosis of ILD, and increased immunosuppression [66].

Rituximab (RTX) is a monoclonal antibody targeting the B-cell marker CD20 and has been approved for treating RA in anti-TNF nonresponders. Follicular B-cell hyperplasia and interstitial plasma cell infiltrates were found in RA-ILD patients. This raised the possibility that B cells were involved in the disease's etiology and aroused interest in using RTX for the treatment of RA-ILD [67]. In a retrospective multicenter cohort study done in Portugal, RTX seems to be a promising treatment for CTD-ILD patients (61.2% of them were RA patients). Impressive efficacy outcomes were observed in patients with non-specific interstitial pneumonia patterns [68]. On the other hand, a meta-analysis of biological treatments for CTD showed that RTX use is linked to an increase in non-infectious parenchymal lung disease [69].

IL-6 inhibitors such as tocilizumab demonstrated that the profibrotic effects of the proinflammatory cytokine IL-6 are countered by IL-6R inhibition [70], indicating a possible advantage of this therapeutic strategy in pulmonary fibrosis caused by RA. In a case series of four RA patients, it has been observed that tocilizumab (TCZ) as monotherapy maintains or potentially improves ILD [71]. In contrast, some have reported ILD incidence or progression after using tocilizumab [59].

In clinical trials, Janus kinase inhibitors (JAKi), particularly tofacitinib (TOF), have demonstrated encouraging outcomes. According to a multicenter retrospective study, JAKi therapy may be a safe therapeutic option for patients with RA-ILD, leading to significant stability of ILD at HRCT and potentially preventing PFT deterioration [72]. Another prospective study done in 2023 with a total of 28,559 patients with RA found that out of all the patients who received bDMARDs, those on tofacitinib had the lowest incidence of ILD [73].

The anti-fibrotic agents might prevent the disease progression of RA-ILD. At the current time, pirfenidone and nintedanib are the two anti-fibrotics that are being studied for the treatment of IPF. The INBUILD research compared nintedanib to a placebo in patients with progressive, fibrotic lung disease (13% RA-ILD), in 15 different countries. The annual rate of reduction in the forced expiratory volume (FVC) was considerably lower in patients receiving nintedanib than in patients receiving a placebo in those with progressive fibrosing ILD. One prevalent adverse reaction was diarrhea [74]. Pirfenidone appeared to be effective in the unclassifiable ILD study, a phase 2 randomized controlled trial that enrolled 253 patients with progressive fibrosing unclassifiable ILD; FVC decreased by 87.7 mL in the treatment arm compared to 157.1 mL with placebo [75]. A summary of the treatment is included in Table 3.

**Table 3:** Overview of the included studies. MTX: methotrexate; RA-ILD: rheumatoid arthritis-associated interstitial lung disease; ILD: interstitial lung disease; CTD-ILD: connective tissue disease-interstitial lung disease; DMARDs: disease-modifying anti-rheumatic drugs; anti-TNF: anti-tumor necrosis factor; JAKi: Janus kinases inhibitors.

Reference	Publication year	Study design	Conclusion summary
Atienza-Mateo et al. [64]	2024	Observational longitudinal study	Early administration of abatacept may prevent RA-ILD progression.
Baker et al. [73]	2023	Retrospective study	Patients treated with tofacitinib had the lowest incidence of ILD compared with patients treated with biological* *DMARDs.
Venerito et al. [72]	2023	Retrospective study	JAKi therapy might be a safe therapeutic option for patients with RA-ILD.
Maher et al. [75]	2022	Placebo-controlled phase 2 trial	Patients with progressive fibrosing unclassifiable ILD could benefit from pirfenidone treatment.
Flaherty et al. [74]	2019	Placebo-controlled phase 3 trial	The annual rate of reduction in the forced expiratory volume was considerably lower in patients receiving nintedanib.
Kiely et al. [49]	2019	Prospective cohort	Treatment with MTX was not linked to a higher incidence of RA-ILD.
Fu et al. [44]	2019	Retrospective cohort	Treatment with cyclophosphamide contributes to improving the prognosis of RA-ILD.
Manfredi et al. [71]	2018	Case series	Tocilizumab (TCZ) as a monotherapy was reported to stabilize or even improve ILD.
Yamano et al. [39]	2018	Retrospective case series	Two courses of pulse-dose methylprednisolone therapy followed by prednisone and oral tacrolimus therapy seemed to be well tolerated and effective.
Rojas-Serrano et al. [50]	2017	-	Treatment with methotrexate was strongly associated with increased survival in RA-ILD patients.
Oldham et al. [45]	2016	Retrospective cohort	Azathioprine use in CTD-ILD was associated with stability in pulmonary function.
Fischer et al. [42]	2013	Retrospective study	Treatment with mycophenolate was associated with either stable or improved pulmonary physiology in CTD-ILD patients.
Dixon et al. [63]	2010	Prospective observational study	When compared to conventional DMARDs, anti-TNF medication does not result in a higher mortality rate in RA-ILD patients.
Douglas et al. [40]	2000	Retrospective study	Patients using colchicine or prednisone did not significantly vary in survival from those receiving no treatment.

Lung transplantation should be considered for patients with severe and progressive RA-ILD who do not have extrapulmonary contraindications and who have not responded to appropriate treatment [76].

Limitations

RA is a complex disease. The frequency and intensity of pulmonary symptoms may differ depending on the severity, duration, coexisting conditions, and the form of lung involvement. This study is primarily focused on the common forms of RA-ILD, such as UIP and NSIP, and did not include enough information about the rare forms.

## Conclusions

Pulmonary involvement in RA can affect any area of the lung, including the pleura, airways, lung parenchyma, and vessels, with varying degrees and frequency. Lung injury mechanisms have been linked to a combination of autoimmune, environmental, and hereditary causes. HRCT is considered the primary diagnostic modality. The improvement of prognosis and quality of life for patients with lung involvement of RA depends on early detection, multidisciplinary cooperation, and tailored therapy approaches. Steroids are typically the first line of treatment, according to the abovementioned studies. After carefully evaluating the patient's prior medical history, further immunosuppressive medications and biologics may be added to the regimen. In the end, additional research is suggested for the future, specifically controlled clinical trials with more patient populations, to develop new and secure treatment alternatives.
